# Validation of the Czech Version of the Relational Needs Satisfaction Scale

**DOI:** 10.3389/fpsyg.2020.00359

**Published:** 2020-03-06

**Authors:** Martina Pourová, Tomáš Řiháček, Gregor Žvelc

**Affiliations:** ^1^Department of Psychology, Faculty of Social Studies, Masaryk University, Brno, Czechia; ^2^Department of Psychology, Faculty of Arts, University of Ljubljana, Ljubljana, Slovenia; ^3^Department of Psychology, UP FAMNIT, University of Primorska, Koper, Slovenia; ^4^Institute for Integrative Psychotherapy and Counselling, Ljubljana, Slovenia

**Keywords:** relational needs satisfaction scale, factor structure, measurement invariance, convergent validity, psychometrics

## Abstract

**Aim:**

If we want to understand people’s satisfaction in their relationships, it is essential to have a valid and reliable measure of relational needs satisfaction. The aim of this study was to test the factor structure of the Czech version of the Relational Needs Satisfaction Scale (RNSS) as well as the scale’s measurement invariance and convergent validity.

**Method:**

In total, 419 adults answered a battery of measures, including the RNSS, in an online survey. Confirmatory factor analysis was conducted to test the factor structure and the measurement invariance of the RNSS across gender and age. A correlational analysis was conducted to assess the convergent validity.

**Results:**

The five-factor structure of the RNSS was confirmed. Furthermore, support for a second-order global relationship satisfaction factor was found. The hierarchical model was strictly invariant with respect to gender and age. Furthermore, the RNSS demonstrated an expected pattern of correlations with the reference instruments.

**Conclusion:**

The Czech version of the RNSS can be considered a valid and reliable method.

## Introduction

Both attachment theory and psychoanalytic theories such as object relations theory and self psychology assert that a child’s basic need is for a relationship with a significant other person (Fairbairn, 1954, 1986/1941; Bowlby, 1969; Kohut, 1971, 1977; Ainsworth et al., 1978; Winnicot, 1986/1960; Guntrip, 1992/1968). Children are dependent on caregivers and need appropriate response to their physical and emotional needs. Successful affective attunement to a child’s emergent needs provides a sense of safety and comfort and is crucial for the development of a child’s secure attachment to a parent or guardian and the development of the child’s sense of self (Stern, 1985; Schore, 1994; Siegel, 1999; Fonagy et al., 2004). On the other hand, chronic misattunement, abuse and neglect of a child may result in an insecure or disorganized attachment and are related to emotional difficulties later in life (Stern, 1985; Schore, 1994, 2001, 2003; Siegel, 1999; Fonagy et al., 2004; Erskine, 2015). The satisfaction of relational needs by another person who is involved and attuned is crucial not only in childhood but also throughout our whole lives (Erskine et al., 1999; Erskine, 2015).

The concept of relational needs was introduced by Richard Erskine and explained in several books and articles (Erskine and Trautmann, 1996; Erskine et al., 1999; Moursund and Erskine, 2004; Erskine, 2015). Erskine’s model of relational needs is widely recognized within the traditions of integrative psychotherapy and transactional analysis. However, the model can be used in different psychotherapy approaches and outside of clinical settings. Erskine (2015) defined relational needs as needs that are “unique to personal contact” (p. 46). Such needs are present in every relationship and define the relationship. These needs are not only needs during childhood but “are present throughout the entire life cycle from early infancy through old age” (Erskine, 2015, p. 47). These needs are the basic components of relationships and are present each day of our lives. Erskine et al. (1999) described eight main relational needs, namely: (1) The need for security, (2) The need to feel validated, affirmed and significant within a relationship, (3) The need for acceptance by a stable, dependable, and protective other person, (4) The need for confirmation of personal experience, (5) The need for self-definition, (6) The need to have an impact on another person, (7) The need to experience initiative from another person, and (8) The need to express love. When relational needs are attended to, a person feels that he or she is being loved. Erskine (2015) describes how a relational need may become conscious through a feeling of longing or desire while the other relational needs may remain out of conscious awareness and in the background. An attuned response by another person to an individual’s relational need meets the relational need, and consequently, the need becomes less intense and recedes into the background of awareness. Relational needs are often out of conscious awareness. However, when relational needs are not attended to, they may become more intense and pressing. The lack of satisfaction of relational needs may be experienced as an “emptiness, a longing, or a nagging loneliness” (Erskine et al., 1999, p. 122). If relational needs are continually dissatisfied, the person may experience frustration and anger, leading to a loss of energy and hope. The continued dissatisfaction of relational needs may be manifested in negative script beliefs about the self, others, and life (Erskine and Moursand, 1988). Such beliefs may include: “I am not worthy of love” or “Life is meaningless.”

Žvelc et al. (unpublished) developed the Relational Needs Satisfaction Scale (RNSS) based on Erskine’ model of relational needs (Erskine et al., 1999; Erskine, 2015). The development of the instrument proceeded through several phases. In the first phase, the authors created 269 items referring to Erskine’s description of eight relational needs. Items were evaluated by five experts in terms of the theoretical representation of the construct, as well as simplicity and clarity, and reduced to 119 items. In the next phase of development, items were tested on two separate samples and the scale was further reduced to 20 items. Through the means of principal component analysis, the authors found five dimensions of relational needs named as Authenticity, Support and Protection, Having an Impact, Shared Experience, and Initiative from the Other (Žvelc et al., unpublished).

The Authenticity dimension describes the need to be authentic in relationships with others (Žvelc et al., unpublished). The satisfaction of this need shows in a person experiencing that he or she can be with others as he or she truly is. This is demonstrated in feelings of security, understanding, and respect by others. A person who has this need satisfied experiences other people accepting his or her uniqueness or individuality. Žvelc et al. (unpublished) relate the need for authenticity to Erskine’s description of the needs for security, validation, and self-definition in a relationship (Erskine et al., 1999; Erskine, 2015). Item examples: “I feel free to show my feelings to others and speak my mind because I know they accept me for who I am”; “I do not have to pretend with people who are important to me.”

The second dimension of relational needs is Support and Protection (Žvelc et al., unpublished). This dimension is related to the experience that a person has someone in his or her life who can be asked for help, protection, and support when in distress. This relational need is related to Erskine’s need for acceptance by a stable, dependable, and a protective other person (Erskine et al., 1999; Erskine, 2015). People who have this relational need satisfied can rely on someone who is strong and supportive. Item examples: “I have at least one person in my life who encourages me, protects me or provides me with the information I need”; “I have a strong, stable and protective person in my life whom I can rely on.”

The dimension Having an Impact refers to the need of a person to feel that he or she has an impact on others (Žvelc et al., unpublished). The satisfaction of this need is related to the experience that other people accept a person’s opinion, advice, or ideas. The person feels that he or she can affect other people and provoke a change in them. This need is related to Erskine’s need to have an impact on another person (Erskine et al., 1999; Erskine, 2015). Item examples: “I feel that I have an influence on others”; “Others often take my advice to heart.”

The dimension Shared Experience describes the experience of a person having people in his or her life with whom he or she shares similar interests and experiences (Žvelc et al., unpublished). People who have this need satisfied, have someone in their lives who experiences something similar and has some similar qualities. This dimension is related to Erskine’s need for confirmation of personal experience (Erskine et al., 1999; Erskine, 2015). Item examples: “There are people in my life with whom I share similar experiences”; “I know people who experience some things similarly to me.”

The dimension Initiative from the Other describes the experience of other people sometimes surprising and helping us without us having to ask for it. This dimension is related to feeling that another person does something for us without our request or demand. The dimension is related to Erskine’s description of the need for the initiative from another person (Erskine et al., 1999; Erskine, 2015). Item examples: “Other people often help me even if I do not specifically ask them to”; “Other people sometimes surprise me in a nice way.”

Žvelc et al. (unpublished) have found empirical support for four of Erskine’s original dimensions of relational needs. They have also found a new dimension called Authenticity, which includes items related to Erskine’s needs for security, validation, and self-definition. Erskine’s Need to Express Love was not found as a separate relational need and, therefore, was not included in the RNSS.

Confirmatory factor analysis was used to test the fit of the instrument regarding two theoretical models: a five-factor model with correlated factors and a hierarchical model with five first-order factors and one second-order factor (Žvelc et al., unpublished). Confirmatory factor analysis showed that the RNSS had a good fit in case of both models. The authors proposed that the hierarchical model is more congruent with the theory of relational needs, since it included the general dimension of relational needs satisfaction (Žvelc et al., unpublished).

The final version of the RNSS includes 20 items representing the five empirical dimensions of relational needs satisfaction. The overall score can also be computed to assess the general satisfaction of relational needs. The RNSS has acceptable to excellent internal reliability. On a sample of 354 participants, Žvelc et al. (unpublished) found the following Cronbach’s alpha coefficients: Authenticity (α = 0.80), Support and Protection (α = 0.85), Having an Impact (α = 0.81), Shared Experience (α = 0.73), and Initiative from the Other (α = 0. 83). The reliability of the overall score was α = 0.90. The authors of the scale also investigated the convergent validity of the instrument. Higher satisfaction of relational needs was related to attachment security, higher self-compassion, emotional well-being, and satisfaction in life (Žvelc et al., unpublished). In a recent study, Grgurić and Žvelc (unpublished) found that lower satisfaction of relational needs was related to a higher score on the measure of internet addiction.

Žvelc et al. (unpublished) proposed that RNSS should be tested on other samples and cross-culturally to further assess the factor validity and stability of the instrument. The aim of the present study was to validate the Czech version of the RNSS. The factor structure and its measurement invariance across age and gender were tested by confirmatory factor analysis. Furthermore, the satisfaction of relational needs was expected to be related to (though not identical with) general distress, well-being, and attachment dimensions. Therefore, convergent validity with measures of general distress, well-being, and attachment was assessed.

## Materials and Methods

### Study Design and Sample

Informed consent was obtained from all individual participants included in the study. The project was approved by the Research Ethics Committee of Masaryk University (ref. no. EKV-2017-029-R1). An online survey was created in the Czech language using the LimeSurvey platform (LimeSurvey Project Team, 2015) hosted at Masaryk University. A link to the survey was spread via social media networks. The link was the same for all participants, allowing them to share it in a snowball manner.

The original sample consisted of 733 adults who opened the survey. However, 305 respondents did not finish the survey and were removed from the sample. The final sample consisted of 428 adults. The sample characteristics are reported in Table 1.

**TABLE 1:** Sample characteristics (*N* = 428).

**Age (years)**	
Mean (*SD*)	30.71 (11.1)
Median	28
Range	18-75
**Gender**	
Female	266(62%)
Male	162(38%)
**Household**	
In partnership	173(41%)
Single	89(21%)
With parents	113(26%)
Other	53(12%)
**Siblings**	
Have siblings	357(83%)
**Marital status**	
Single	268(63%)
Married	99(23%)
Divorced	34(8%)
Widowed	3(1%)
Other	24(6%)
**Children**	
Have own children	131(31%)
**Education**	
Primary school	46(11%)
Secondary school	210(49%)
University	172(40%)
**Nationality**	
Czech	414(97%)
Slovak	9(2%)
Other	5(1%)
**Occupation**	
Employe	195(46%)
Entrepreneur	42(10%)
Unemployment	6(2%)
Maternity leave	30(7%)
Student	142(33%)
Retirement	2(0%)
Invalidity pension	1(0%)
Other	10(2%)

### Measures

#### Relational Needs Satisfaction Scale

The RNSS (Žvelc and Jovanoska, unpublished; Žvelc et al., unpublished) is a self-report questionnaire. It consists of 20 items devised to measure five theoretical dimensions of relational needs, namely: Support and protection, Having an Impact, Authenticity, Shared Experience, and Initiative from the Other (Žvelc et al., unpublished). Each subscale comprises 4 items. Each item is rated on a 5-level scale, where 1 means ‘never true’ and 5 means ‘always true.’ Subscale scores are computed as a mean of the respective item scores (Žvelc and Jovanoska, unpublished). The overall score is computed as a grand mean of all items. A higher score reflects a higher level of satisfaction in the particular dimension.

The scale was translated into Czech from the English version. Five independent Czech translations were made by native Czech speakers (a psychology student, two psychologists, and two laypeople). Second, all translations were discussed by a group of three people (the two psychologists and the psychology student) and consolidated into a single version. Third, this version was back-translated into English by a bilingual, native English speaker and compared to the original English version. Fourth, the back-translation was discussed with the authors of the scale, and minor corrections were made based on this discussion. Fifth, the final Czech version was field-tested with five respondents to check the comprehensibility of the items.

#### Experiences in Close Relationships – Relationships Structure

The ECR-RS (Fraley et al., 2011, 2015) is a self-report questionnaire designed to assess attachment patterns in a specific relationship (i.e., mother, father, romantic partner, or best friend) or global attachment (i.e., feelings about close relationships in general). The global attachment version was used in this study since the RNSS is focused on respondents’ general relational experience as well. The scale consists of 9 items and measures two attachment dimensions, global avoidance (items 1 to 6) and global anxiety (items 7 to 9). Each item is rated on a 7-point scale, where 1 means “strongly disagree” and 7 means “strongly agree.” Four items are reverse-keyed. Global avoidance and global anxiety scores are computed as averages of the respective items. The two-factor structure was supported by other studies as well, albeit some items tended to cross-load, and the scales had good internal reliability (Feddern Donbaek and Elklit, 2014; Moreira et al., 2015). The Czech version of the scale (Cígler et al., 2019) was used in the study, which has demonstrated similar psychometric properties as the English version. In our sample, the internal consistency was α = 0.85 for global avoidance and α = 0.89 for global anxiety; the correlation between the two subscales was *r* = 0.19. Since the perception of relationships is related to the attachment style (Fraley et al., 2015), we expected to find a correlation between the two instruments.

#### Well-Being Index (WHO-5)

The WHO-5 (Bech et al., 2003) is a unidimensional self-report measure of well-being. The scale has good to excellent internal consistency (Krieger et al., 2014). The unidimensionality of the scale was validated across different languages and populations and it was demonstrated to be a sensitive and specific screening tool for depression (Topp et al., 2015). It consists of 5 items rated from 0 to 5, where 5 means “all the time” and 0 means “never”(Bech et al., 2003). The Czech version of the scale was used (WHO Collaborating Center for Mental Health, 1998). The internal consistency in this study was α = 0.86. Since the satisfaction of relational needs is related to subjective well-being (Soons and Liefbroer, 2008), we expected to find a correlation between the two instruments.

#### Clinical Outcome Measure in Routine Evaluation – General Population (GP-CORE)

The GP-CORE (Barkham et al., 2001) is a self-report measure of subjective distress designed for the general population. It consists of 14 items. The scale has a good internal consistency. Although four components were found empirically, namely positive and negative subjective well-being, physical problems, and social functioning, the respective subscale scores tend to be highly correlated, and the authors recommended that the measure be used as unidimensional (Evans et al., 2005). Each item is rated on a 5-point scale, where 0 means “not at all” and 4 means “most or all the time.” Eight items are reverse-keyed (Barkham et al., 2001). The Czech version of the measure was used (Juhová et al., 2018). The internal consistency of the overall score in this study was α = 0.88. Since the dissatisfaction of relational needs is related to higher subjective distress (Frey et al., 2004), we expected to find a correlation between the two instruments.

#### Demographic Questionnaire

The demographic questionnaire contained questions about respondents’ gender, age, nationality, education, occupation, and marital status. Furthermore, respondents were asked if they had siblings and/or children of their own.

### Statistical Analysis

To test the factor structure of the RNSS, we conducted confirmatory factor analysis (CFA). Since the values of some RNSS items were non-normally distributed, we used the robust maximum likelihood (MLR) estimator. All models were defined as congeneric (i.e., without any cross-loading items and without any further restriction). The metric of each latent variable was based on its first indicator.

Model fit was assessed using scaled chi-square statistics, standardized root mean square residual (SRMR), root mean square error of approximation (RMSEA), and the Tucker-Lewis Index (TLI). Hu and Bentler (1999) recommend values close to 0.08 for SRMR, 0.06 for RMSEA, and 0.95 for TLI as cutoffs for a fitting solution. Other authors, however, have suggested less stringent criteria for model rejection: RMSEA > 0.10 and TLI < 0.90 (see Brown, 2015). Internal consistency of the individual factors was assessed using Cronbach’s alpha and McDonald’s omega. A *post hoc* power analysis was conducted to determine the statistical power to detect a model with RMSEA ≥ 0.06 at α = 0.05.

As an auxiliary method to explore the patterns of factor scores in the sample, cluster analysis was used. The partitioning around medoids clustering method was used, and the number of clusters was estimated using the optimum average silhouette width (Reynolds et al., 2006).

Furthermore, we tested measurement invariance (a) between women and men and (b) across two age groups (the sample was split based on the median value). We gradually fixed factor loadings (metric invariance), item intercepts (scalar invariance), and residual variances (strict invariance). Invariance was assessed by a change in fit compared to a previous model: a change in TLI ≥ 0.010 (for all levels of invariance), supplemented by a change in RMSEA ≥ 0.015 (for all levels of invariance) or a change in SRMR ≥ 0.030 (for metric invariance) and ≥ 0.010 (for scalar and strict invariance), which indicate non-invariance in samples with *N* > 300 (Chen, 2007). We also conducted a significant difference test using Satorra and Bentler’s (2010) approach. If the assumption of strict invariance was found tenable, we compared latent means (i.e., intercepts) across groups and reported standardized effect sizes (Cohen’s *d*).

To test the convergent validity of the RNSS, we assessed the associations of the RNSS scores with three reference instruments (ECR-RS, GP-CORE, and WHO-5). We strived to choose reference instruments that were as similar as possible to those used in the original study (Žvelc et al., unpublished) and that were available in Czech. Since some of the variables were non-normally distributed, we used the Spearman correlation coefficient. For informative purposes, we also conducted a CFA for each reference instrument, testing unidimensional models for the GP-CORE and WHO-5 and a two-factor model for the ECR-RS.

To assess the common method variance, we conducted Harman’s single factor test (Podsakoff et al., 2003). We pooled all items of the RNSS, ECR-RS, GP-CORE, and WHO-5 and conducted an exploratory factor analysis (EFA) using the minimum residual factoring method for factor extraction. We examined the unrotated solution. The number of factors was determined using Horn’s parallel analysis, scree plot, and Kaiser’s rule. Common method variance is considered problematic if either a single factor emerges or one factor accounts for the majority of the covariance among items. The analysis was conducted using R version 3.4.4 (R Core Team, 2017), with the lavaan (Rosseel, 2012), semTools (semTools Contributors, 2016), semPlot (Epskamp and Stuber, 2017), cluster (Maechler et al., 2016), fpc (Hennig, 2015), psych (Revelle, 2018), nFactors (Raiche, 2010), and semPower (Moshagen, 2018) packages. The data (“data.xlsx”) and R script (“rnss.r”) are available in the Open Science Framework (Pourová and Øiháèek, 2019).

## Results

### Missing Data

Nine cases provided incomplete responses to the questionnaire and were not included in the confirmatory factor analysis. Therefore, we proceeded with a sample of *N* = 419. For the purpose of convergent validity testing, five cases with more than one missing item per measure were removed, resulting in 423 adults who completed the survey.

### Confirmatory Factor Analysis

We tested the original five-factor model (Model 1). The model demonstrated a decent fit and could be accepted. However, the latent variables were highly inter-correlated, which suggested either the existence of a higher-order latent variable or the existence of a unidimensional structure.

To test the first possibility, we specified a hierarchical model in which all first-order factors loaded on a single second-order factor (Model 2). Although the chi-square test suggested a drop in fit, the deterioration of other fit indices was negligible, and the model demonstrated a good fit.

**FIGURE 1:**
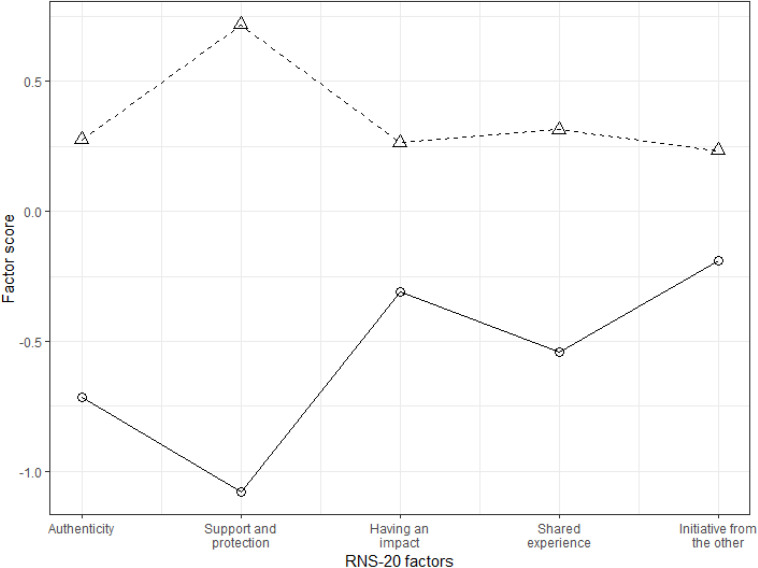
Clusters based on the satisfaction of relational needs.

To determine whether a more parsimonious, unidimensional structure was adequate, we conducted cluster analysis based on the estimated factor scores of each respondent. Two clusters were identified, one with respondents scoring low on all latent variables and the other with respondents scoring high on all of them (see Figure 1). This result was interpreted as support for a unidimensional latent structure of the measure, and therefore, we proceeded with testing the unidimensional model (Model 3). However, this model demonstrated a considerable lack of fit and could not be accepted.

**FIGURE 2:**
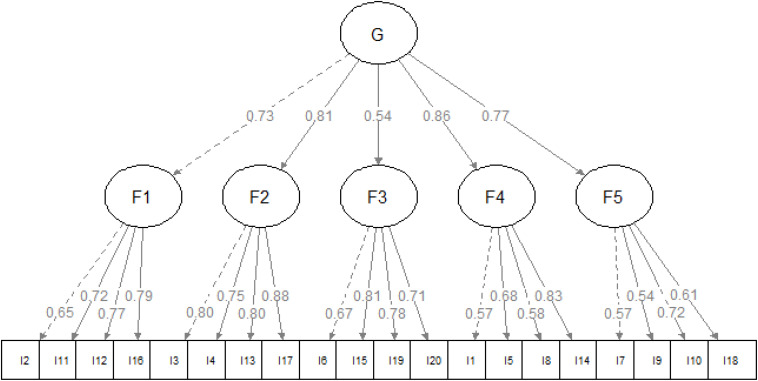
The hierarchical model, χ2(165) = 317.656, *p* < 0.001, BIC = 21537.633, SRMR = 0.05, RMSEA = 0.051, TLI = 0.942.

MLR estimation for all models converged successfully. See Table 2 for factor loadings and correlations and Table 3 for fit indices. The RMSEA of the null model was 0.212. Although Model 1 had the best fit, we considered the hierarchical model (Model 2) the most appropriate one since it accounted for the covariance among the first-order factors and it was also a more parsimonious solution in terms of the number of estimated parameters. The model is plotted in Figure 2. The *post hoc* power analysis revealed the statistical power of (1 – β) > 0.9999.

**TABLE 2:** Factor loadings and correlations (completely standardized).

Items	Model 1 (five-factor)						Model 2 (hierarchical)						Model 3 (unidimensional)	
	λ_1_	λ_2_	λ_3_	λ_4_	λ_5_	ε	λ_1_	λ_2_	λ_3_	λ_4_	λ_5_	ε	λ	ε
I2	0.648					0.580	0.649					0.579	0.573	0.671
I11	0.717					0.487	0.718					0.484	0.528	0.721
I12	0.771					0.406	0.771					0.405	0.547	0.701
I16	0.794					0.369	0.792					0.373	0.647	0.582
I3		0.800				0.360		0.801				0.359	0.690	0.524
I4		0.746				0.443		0.749				0.439	0.686	0.530
I13		0.804				0.354		0.804				0.353	0.736	0.458
I17		0.885				0.216		0.883				0.221	0.801	0.358
I6			0.662			0.561			0.671			0.550	0.383	0.853
I15			0.810			0.343			0.811			0.342	0.495	0.755
I19			0.787			0.381			0.782			0.389	0.392	0.847
I20			0.713			0.492			0.710			0.495	0.405	0.836
I1				0.563		0.683				0.565		0.680	0.452	0.795
I5				0.674		0.545				0.676		0.534	0.566	0.680
I8				0.582		0.662				0.582		0.661	0.501	0.749
I14				0.836		0.302				0.833		0.306	0.730	0.467
I7					0.574	0.671					0.574	0.670	0.438	0.808
I9					0.521	0.729					0.539	0.709	0.391	0.847
I10					0.728	0.470					0.717	0.486	0.552	0.695
I18					0.618	0.619					0.615	0.622	0.509	0.741

**Factor correlations**							**Second-order factor loadings**							

F1		0.607	0.466	0.623	0.507		0.732					0.465		
F2			0.338	0.699	0.676		0.813					0.339		
F3				0.514	0.419		0.538					0.711		
F4					0.640		0.862					0.256		
F5							0.769					0.409		

**TABLE 3:** Fit indices for the tested models (*N* = 428).

Model	χ*^2^*	*df*	*Δ*χ*^2^*	*Δdf*	BIC	SRMR	*Δ*SRMR	RMSEA [90% CI]	*Δ*RMSEA	TLI	*Δ*TLI
**Hypothesized models**											
Model 1 (Five-factor)	298.915***	160			21544.658	0.043		0.049 [0.040;0.058]		0.946	
Model 2 (Hierarchical)	317.656***	165	17.680**	5	21537.633	0.047	−0.004	0.051 [0.042;0.059]	−0.002	0.942	−0.004
Model 3 (Unidimensional)	1187.974***	170	963.730***	5	22514.683	0.095	−0.048	0.129 [0.122;0.136]	−0.078	0.628	−0.314

BIC, Bayesian Information Criterion; SRMR, Standardized Root Mean Square Residual; RMSEA, Root Mean Square Error of Approximation with 90% confidence intervals; TLI, Tucker-Lewis Index. Robust estimates of fit indices are reported. ** *p* < 0.01, *** *p* < 0.001.

The internal consistency was good to acceptable for the five subscales: α = 0.82 (ω_h_ = 0.82) for Authenticity, α = 0.88 (ω_h_ = 0.88) for Support and Protection, α = 0.83 (ω_h_ = 0.84) for Having an Impact, α = 0.77 (ω_h_ = 0.75) for Shared Experience and α = 0.70 (ω_h_ = 0.71) for Initiative from the Other. Furthermore, the internal consistency of the second-order factor was ω = 0.81 at Level 1 (i.e., the proportion of the second-order factor explaining the total score) and ω = 0.86 at Level 2 (i.e., the proportion of the second-order factor explaining the variance at the first-order factor level). The Cronbach’s alpha of the overall scale was α = 0.90 (ω_h_ = 0.94). Thus, the internal consistency of the overall scale can be considered excellent.

### Measurement Invariance Across Gender and Age

The measurement invariance for the hierarchical model was tested (see Table 4 for fit indices). First, we tested invariance with respect to gender. Although the chi-square difference was significant for the scalar and strict levels, other fit indices suggested that the model was strictly invariant with respect to gender. Women scored higher than men on the second-order factor (the standardized difference in latent means was *d* = 0.40, *p* = 0.000) and on Having an Impact (*d* = 0.28, *p* = 0.000), while men scored higher on Authenticity (*d* = −0.22, *p* = 0.029). The differences between men and women were non-significant for Support and Protection (*d* = −0.09, *p* = 0.286), Shared Experience (*d* = 0.14, *p* = 0.137), and Initiative from the Other (*d* = −0.11, *p* = 0.200).

**TABLE 4:** Fit indices for invariance testing.

Invariance	χ*^2^*	*df*	*Δ*χ*^2^*	*Δdf*	BIC	SRMR	*Δ*SRMR	RMSEA	*Δ*RMSEA	TLI	*Δ*TLI
**Gender**											
Configural	524.198***	330			21770.93	0.058		0.056 [0.047;0.065]		0.928	
Metric	548.392***	349	24.194	19	21683.62	0.068	−0.010	0.056 [0.047;0.064]	−0.001	0.930	−0.002
Scalar	580.141***	363	31.746**	14	21633.23	0.070	−0.002	0.057 [0.048;0.065]	−0.001	0.927	0.003
Strict	621.609***	383	41.468**	20	21559.01	0.072	−0.002	0.058 [0.050;0.066]	−0.001	0.924	0.003
Means	656.777***	389	35.169***	6	21559.51	0.083	−0.011	0.061 [0.053;0.069]	−0.003	0.916	0.008
**Age**											
Configural	501.844***	330			21728.91	0.058		0.053 [0.043;0.062]		0.938	
Metric	541.120***	349	39.276**	19	21659.64	0.076	−0.018	0.055 [0.045;0.063]	−0.002	0.935	0.004
Scalar	610.981***	363	69.861***	14	21653.34	0.079	−0.003	0.061 [0.052;0.069]	−0.006	0.919	0.016
Strict	649.720***	383	38.740**	20	21575.72	0.079	0.000	0.061 [0.053;0.069]	−0.001	0.918	0.001
Means	676.740***	389	27.019***	6	21567.36	0.081	−0.002	0.063 [0.055;0.071]	−0.002	0.913	0.005

BIC, Bayesian Information Criterion; SRMR, Standardized Root Mean Square Residual; RMSEA, Root Mean Square Error of Approximation with 90% confidence intervals; TLI, Tucker-Lewis Index. *** *p* < 0.001, ** *p* < 0.01.

Second, we tested invariance between two age groups. The sample was split according to the median value (age ≤ 28 vs. age > 28). Although the chi-square difference was significant for all levels of invariance, other fit indices suggested that the model was strictly invariant with respect to age. Younger people scored higher than older on Having an Impact (*d* = 0.19, *p* = 0.000) and Shared Experience (*d* = 0.16, *p* = 0.043) and lower on Initiative from the Other (*d* = −0.29, *p* = 0.001). The differences between the age groups were non-significant for Support and Protection (*d* = −0.12, *p* = 0.115), Authenticity (*d* = −0.16, *p* = 0.090), and for the second-order factor (*d* = 0.03, *p* = 0.795).

### Convergent Validity

We tested the associations between the RNSS and three reference instruments (ECR-RS, GP-CORE, and WHO-5). See Table 5 for the results. Generally, the correlations tended to be medium in size, all in the expected direction. The associations of the RNSS subscales with global anxiety scores are somewhat lower than those with global avoidance scores, suggesting that attachment avoidance is more closely related to relational needs satisfaction than attachment anxiety is. The measures of distress and well-being were meaningfully related to all of the RNSS subscales, as well as to the overall score.

**TABLE 5:** Convergent validity of RNSS.

	Authenticity	Support and protection	Having an impact	Shared experience	Initiative from the other	Overall score
GP-CORE	−0.56	−0.47	−0.38	−0.44	−0.37	−0.61
WHO-5	0.48	0.37	0.36	0.37	0.32	0.51
ECR-RS: Global avoidance	−0.50	−0.48	−0.27	−0.39	−0.40	−0.58
ECR-RS: Global anxiety	−0.36	−0.21	−0.28	−0.23	−0.32	−0.39

*RNSS, Relational Needs Satisfaction Scale; ECR-RS, Experiences in Close Relationships – Relationships Structure; WHO-5, Well-Being Index; GP-CORE, Clinical Outcome Measure in Routine Evaluation – General Population; *N* = 423. All correlations were significant at *p* < 0.001.*

Furthermore, we conducted Harman’s single factor test. Parallel analysis, Kaiser’s criterion, and the scree plot converged on a ten-factor solution. Within this solution, the first factor accounted for 29% of variance and, therefore, we concluded that common method variance did not pose a problem in our analysis.

We also tested the expected measurement models for the reference instruments. For the ECR-RS, χ^2^(26) = 194.468, *p* < 0.001, SRMR = 0.088, RMSEA = 0.132 [90%-CI: 0.115; 0.149], TLI = 0.875. For the GP-CORE, χ^2^(77) = 451.034, *p* < 0.001, SRMR = 0.073, RMSEA = 0.116 [90%-CI: 0.106; 0.126], TLI = 0.805. For the WHO-5, χ^2^(5) = 39.304, *p* < 0.001, SRMR = 0.039, RMSEA = 0.146 [90%-CI: 0.105; 0.189], TLI = 0.914. Although the fit was suboptimal for these measures, we did not proceed with further analyses, since the primary focus of this study was on the RNSS.

## Discussion

The aim of this study was to test the factorial structure of the Czech adaptation of the Relational Needs Satisfaction Scale (RNSS) on a general population sample. We found support for both the five-factor structure and the hierarchical model as proposed by the authors of the scale (Žvelc et al., unpublished). We concluded that a hierarchical model with one second-order factor was more appropriate since it accounted for the covariance among the five factors. This is congruent with Žvelc’s et al. (unpublished) conclusion that the hierarchical model is also more congruent with the theoretical background of the scale.

While the existence of a higher-order factor suggests an underlying unidimensionality of the scale, the results were somewhat contradictory. On the one hand, a cluster analysis supported the existence of a single global construct of relational needs satisfaction since the respondents tended to score either high or low on all subscales. On the other hand, a unidimensional model performed poorly in CFA and could not be accepted. We conclude that although the five factors can be clearly distinguished in terms of the factor structure, they failed to demonstrate unique patterns of relationships compared to other instruments. Therefore, future studies should explore their differential relationships with other constructs—in terms of concurrent and predictive validity—to justify their existence as separate subscales. At the same time, our study lends support to the use of the RNSS overall score as an index of the global satisfaction of one’s relational needs that has excellent internal consistency.

Our results demonstrated strict measurement invariance across gender. Thus, the RNSS measures the same construct in both men and women and may be safely used to compare men’s and women’s scores. In our sample, women scored significantly higher than men on the second-order factor. This result suggests that there is a difference in the level of perceived global satisfaction in relationships between men and women. This result is consistent with previous research (e.g., Buunk and VanYperen, 1989; Andersen et al., 1995; Vangelisti and Daly, 1997; Soons and Liefbroer, 2008; Sprecher et al., 2016). While it may mean that women’s needs are more satisfied in relationships than those of men, it may also indicate that both of these genders differ in their expectations about relationships and assess their satisfaction in a different framework based on their gender-role experience (Vangelisti and Daly, 1997).

Interestingly, women scored higher than men on the Having an Impact factor. This contradicts the traditional view of men as more dominant and, therefore, more influential (Carli, 2001). However, as Eagly (1983) observed, these differences may stem largely from inequalities in formal status: men are more likely to have high-status roles, which gives them an opportunity to exert more influence. In intimate relationships, an interpersonal impact can be realized in many ways. While women feel less influential when using dominant forms of communication (Carli, 2001), they may influence another person through care, responsibility, and advice.

Furthermore, men scored higher on the Authenticity factor. While this finding corresponds to a stereotypical representation of men as more straightforward and women as more diplomatic in relationships, it is not consistent with previous research (Brunell et al., 2010). Neff and Suizzo (2006) offer a more complex framework to understand gender differences in authenticity. They build on Cross and Madson’s (1997) theory, which postulates that men construct themselves as independent, whereas women construct themselves as interdependent. Consequently, Neff and Suizzo (2006) suggest that men feel more authentic than women do when they are dominant in their relationships since dominance is an important part of an independent self-concept. Analogically, women may feel more authentic in a subordinate position since meeting the needs of others is an important part of an interdependent self-concept.

We also demonstrated strict measurement invariance across the two age groups as split by the median. Unfortunately, the sample size and age distribution did not allow us to systematically test the invariance across several age-defined cohorts to draw stronger conclusions about measurement invariance across age. Interestingly, there was no significant difference in the second-order factor between the two age groups, suggesting that the global satisfaction of relational needs is equal in younger and older people.

It is worth mentioning that younger people scored higher than older people did on the Having an Impact and Shared Experience factors but lower on the Initiative from the Other factor. These findings can be interpreted in light of a recent study on young Czech adults (Lacinová et al., 2016). The authors found that young adults tend to form intimate relationships without a perspective of the future, focusing on shared here-and-now experiences. Young adults also reported a lower commitment in intimate relationships compared to older adults, which may explain why they experience less initiative from the other person. The higher scores on the Having an Impact factor may reflect the fact that young adults assess their impact in comparison to their previous life in their primary family; after becoming independent from their primary family, they may perceive more opportunities to influence others.

Furthermore, associations of the RNSS scores with three reference instruments were explored to assess the convergent validity of the scale. The pattern of correlations suggested a medium to strong positive relationship with well-being and a negative relationship with actual distress. The analysis also demonstrated that the higher the attachment avoidance or anxiety was, the lower the satisfaction of relational needs was. The associations were somewhat stronger in the case of attachment avoidance; in other words, attachment avoidance predicted the satisfaction of relational needs. The result seems logical: while people with attachment anxiety seek to alleviate their anxiety *through* close relationships, those with attachment avoidance tend to *avoid* close relationships and, consequently, have less opportunity to satisfy their relational needs. Overall, the pattern of correlations resembled the results of the original study (Žvelc et al., unpublished) as well as those of other previous studies (Wongpakaran and Wongpakaran, 2012).

## Limitations and Future Directions

Several limitations are related to the study design and the sample. First, the invitation to participate in this study was disseminated in a snowball manner on social media, and therefore, it was not possible to estimate the number of people who saw the invitation. Furthermore, the study did not implement any safeguards to prevent a recipient from completing the survey more than once.

Second, since the data were collected at one time point, our results could have been affected by the common method variance. However, Harman’s single factor test did not indicate that common method variance would play a fundamental role in our case. Furthermore, the cross-sectional design of the study did not allow us to assess the test-retest reliability of the scale.

Third, social desirability bias could have influenced respondents’ answers to the survey questions. However, since the survey was completely anonymous, there is no reason to believe that this bias would represent a severe threat to the external validity of our study.

Fourth, despite the age range, people above the age of 40 were underrepresented in the sample. Future studies may thus focus on various age groups more systematically. Furthermore, the distribution of the RNSS scores was negatively skewed, which suggests that people with higher levels of relational satisfaction were more likely to respond to our survey compared to those with lower levels of satisfaction. The particularities of our sample and the context of data collection place limits on the generalizability of our findings to other populations and settings. Nevertheless, the whole range of RNSS values was represented in the sample and, therefore, this bias did not pose a serious threat to the internal validity of the study.

Fifth, although the internal consistency was good for all reference instruments, they performed suboptimally in CFA. Therefore, their factor structures should be carefully examined in future studies.

## Conclusion

The Czech version of the Relational Needs Satisfaction Scale (RNSS) is a method for measuring relational needs satisfaction. The scale is based on Erskine’s model of relational needs, which has roots in attachment theory, object relations theory, self-psychology, and transactional analysis. This study supported a hierarchical model of the RNSS and demonstrated its measurement invariance across gender and age. Furthermore, the RNSS demonstrated an expected pattern of correlations with the reference instruments. In conclusion, the Czech version of the RNSS is a valid and reliable measure and can be safely used to compare scores across gender and age groups.

## Data Availability Statement

The raw data supporting the conclusions of this article are available at: https://osf.io/km9ag.

## Ethics Statement

The studies involving human participants were reviewed and approved by Research Ethics Committee of Masaryk University. Written informed consent for participation was not required for this study in accordance with the national legislation and the institutional requirements.

## Author Contributions

MP collected the data, analyzed the data, and wrote the manuscript. TŘ performed the supervision of data analysis and wrote the manuscript. GŽ wrote the manuscript.

## Conflict of Interest

TŘ has received research grant from the Czech Science Foundation. The remaining authors declare that the research was conducted in the absence of any commercial or financial relationships that could be construed as a potential conflict of interest.
