# Corrigendum: Validation of the Czech Version of the Relational Needs Satisfaction Scale

**DOI:** 10.3389/fpsyg.2021.692929

**Published:** 2021-05-12

**Authors:** Martina Pourová, Tomáš Riháček, Gregor Žvelc

**Affiliations:** ^1^Department of Psychology, Faculty of Social Studies, Masaryk University, Brno, Czechia; ^2^Department of Psychology, Faculty of Arts, University of Ljubljana, Ljubljana, Slovenia; ^3^Department of Psychology, UP FAMNIT, University of Primorska, Koper, Slovenia; ^4^Institute for Integrative Psychotherapy and Counselling, Ljubljana, Slovenia

**Keywords:** relational needs satisfaction scale, factor structure, measurement invariance, convergent validity, psychometrics

In the original article, there was a mistake in Table 5 as published. In the published version, the variables in the leftmost column are presented in a wrong order. The correct order is as follows: GP-CORE; WHO-5; ECR-RS - Global avoidance; ECR-RS - Global anxiety. The corrected Table 5 appears below.

**Table 5:** Convergent validity of RNSS.

	**Authenticity**	**Support and protection**	**Having an impact**	**Shared experience**	**Initiative from the other**	**Overall score**
GP-CORE	−0.56	−0.47	−0.38	−0.44	−0.37	−0.61
WHO-5	0.48	0.37	0.36	0.37	0.32	0.51
ECR-RS: Global avoidance	−0.50	−0.48	−0.27	−0.39	−0.40	−0.58
ECR-RS: Global anxiety	−0.36	−0.21	−0.28	−0.23	−0.32	−0.39

*RNSS, Relational Needs Satisfaction Scale; ECR-RS, Experiences in Close Relationships – Relationships Structure; WHO-5, Well-Being Index; GP-CORE, Clinical Outcome Measure in Routine Evaluation – General Population; N = 423. All correlations were significant at p < 0.001*.

The authors apologize for this error and state that this does not change the scientific conclusions of the article in any way. The original article has been updated.

